# Trans Oral Robotic Functional Expansion Pharyngoplasty (TORFEP) with Unidirectional Barbed Sutures

**DOI:** 10.3390/jcm14113904

**Published:** 2025-06-02

**Authors:** Riccardo Nocini

**Affiliations:** Unit of Otolaryngology—Head and Neck Department, University of Verona, P.le L.A. Scuro 10, 37134 Verona, Italy; riccardo.nocini@aovr.veneto.it; Tel.: +39-045-8128332

**Keywords:** pharyngoplasty, TORS, OSAS, robotic surgery

## Abstract

**Background**: Collapse of the lateral pharyngeal wall (LPW) in the pathogenesis of OSA appears to be the only independent risk factor for OSA. Therefore, since 2003, when Cahali first published the technique of lateral pharyngoplasty, several surgical techniques targeting the LPW have been described. Central to these is the concept of widening and stabilizing the pharyngeal airspace by treating the collapse of the LPW rather than removing the redundant pharyngeal soft tissue. The advent of robotic surgery has led to the development of new techniques in OSA surgery, the main target of which is the base of the tongue. Pharyngoplasty using robotic technology can be beneficial when this procedure is combined with tongue base reduction, which is known to be best performed with robotic surgery. **Methods**: This article presents a new technique for functional expansion pharyngoplasty (FEP), which is a modification of the functional expansion pharyngoplasty previously described by Sorrenti and Piccin and is performed using robotic surgery with a Da Vinci system. **Results and Conclusions**: Transoral robotic functional expansion pharyngoplasty is an effective, standardizable technique for treating OSA, notable for its ease of learning and performing.

## 1. Introduction

The mechanism of obstructive sleep apnea (OSA) is usually multifactorial, and upper airway obstruction is most likely multilevel, especially in patients with moderate to severe OSA [1,2,3]. It is therefore difficult to find the correct mechanism underlying the pathology, but it is necessary to provide the correct surgical indication for the treatment of the disease. In fact, the success of the surgical treatment of OSA depends on the accurate diagnosis of the sites where the obstruction occurs and on the appropriate choice of procedures to treat these sites. To this end, several diagnostic considerations have been made in the literature, with drug-induced sleep endoscopy being considered the most objective assessment of the airway during sleep. Based on this premise, the role of the lateral pharyngeal wall (LPW) in the pathogenesis of OSA was demonstrated by Schwab and later by various imaging and drug-induced sleep endoscopic studies [4]. The collapse of the lateral oropharyngeal walls during sleep correlates with the presence and severity of sleep apnea. Based on these results, collapse of the LPW appears to be one of the most important independent risk factors for OSA [5,6]. This led several surgeons to try to find a way to prevent this collapse in order to treat the disease. Consequently, since 2003, when Cahali first published the technique of lateral pharyngoplasty, several surgical techniques targeting the LPW have been described [7]. Central to these is the concept of widening and stabilizing the pharyngeal airspace by treating the collapse of the LPW rather than removing the redundant pharyngeal soft tissue. More recently, with the advent of robotic surgery, several procedures have been developed to treat lesions of the oropharynx, particularly malignant lesions. With increasing use and experience with robotic-assisted transoral surgery (TORS), several authors have attempted to focus on the treatment of even benign conditions such as OSA, particularly procedures aimed at reducing the volume of the base of the tongue in OSA patients [8]. Prior to the advent of TORS, the base of the tongue was rarely treated, as it is a difficult region to expose, and bleeding can be catastrophic. Robotic surgery can limit these difficulties. Another important point is that patients with OSA have multiple levels of obstruction. Therefore, robotic pharyngoplasty may be beneficial when this procedure is combined with tongue base reduction, which is known to be best performed with robotic surgery to maximize the effectiveness of the surgery.

The aim of this article is to present a new technique for functional expansion pharyngoplasty (FEP), which is a modification of the functional expansion pharyngoplasty previously described by Sorrenti and Piccin and is performed with the aid of a robot-assisted procedure using a Da Vinci Xi system [9,10]. This preliminary report outlines the standard of care at our center since 2024. Additional patients are being recruited to further assess the procedure’s efficacy and safety.

This robotic FEP technique entails the splinting of the lateral pharyngeal wall and the advancement of the soft palate. This is accomplished through the superolateral repositioning of the palatopharyngeal muscle, adopting a more physiologic approach to the lateral pharyngeal wall and soft palate. This method aims to both expand the pharyngeal airspace and reduce pharyngeal collapse without compromising the velum muscles, ensuring the avoidance of scarring in the velum.

## 2. Technique

**Figure 1:**
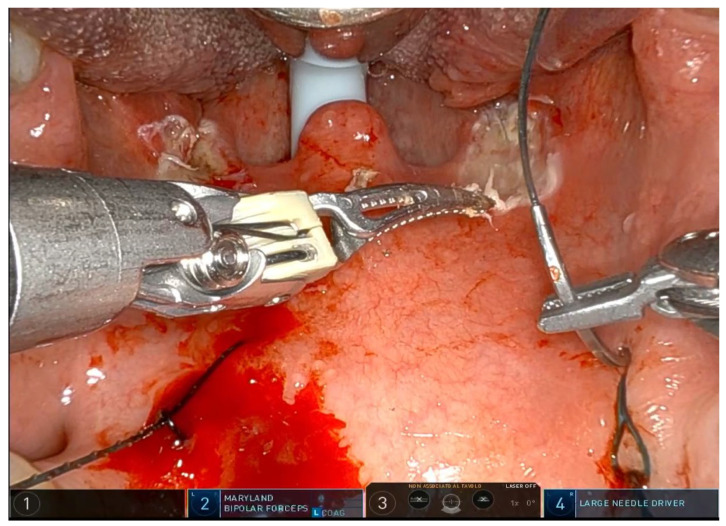
After tonsillectomy the needle is inserted with a self-locking 2-0 barbed suture medial to the apex of the pterygomandibular fold approximately behind the last upper molar, passed around the PMF, taking care to include the periosteum of the maxillary tuberosity, and a U-stitch is made, hooking the end loop of the suture.

**Figure 2:**
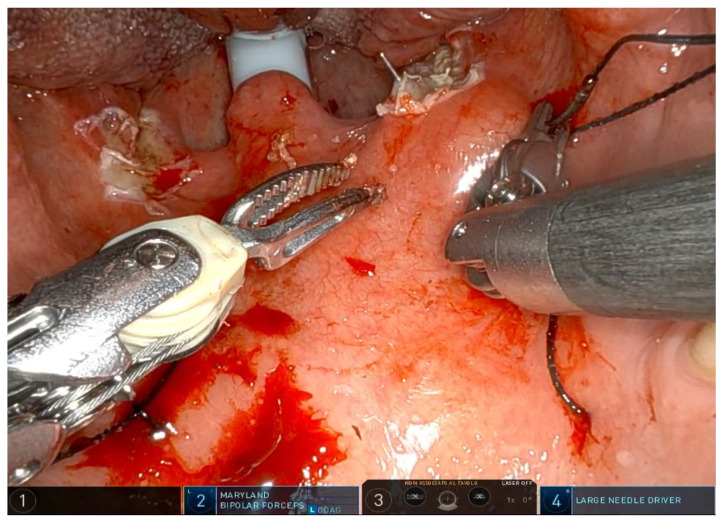
The needle is inserted laterally into the PMF until it exits at the apex of the tonsillar fossa, positioned between the two tonsillar pillars. As shown in Figure 2, it may be necessary to pass the needle twice.

**Figure 3:**
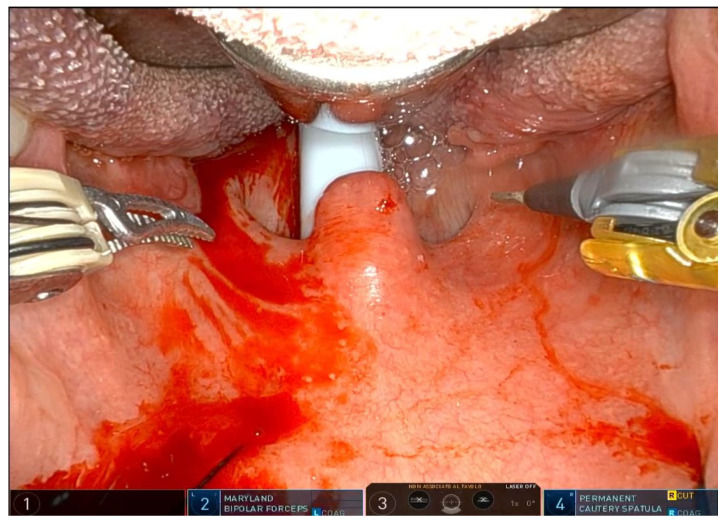
The comparison of the condition immediatley before surgery.

**Figure 4:**
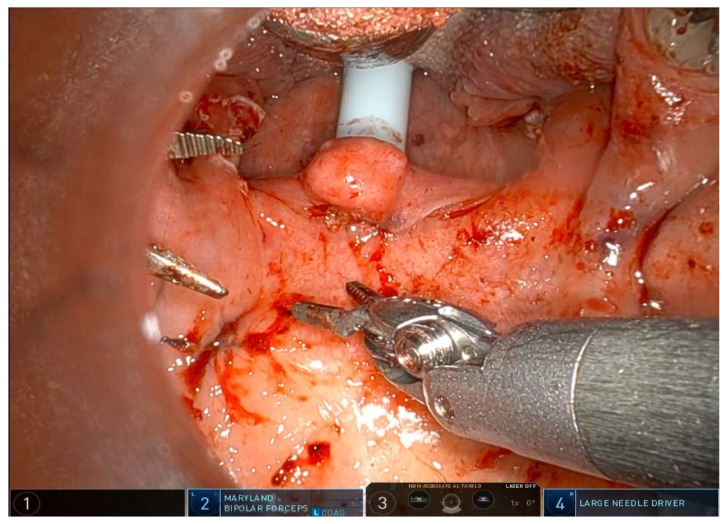
The comparison after all passages have been completed bilaterally shows that the palate is under more tension and is more expanded, which increases the width of the oropharyngeal space.

**Figure 5:**
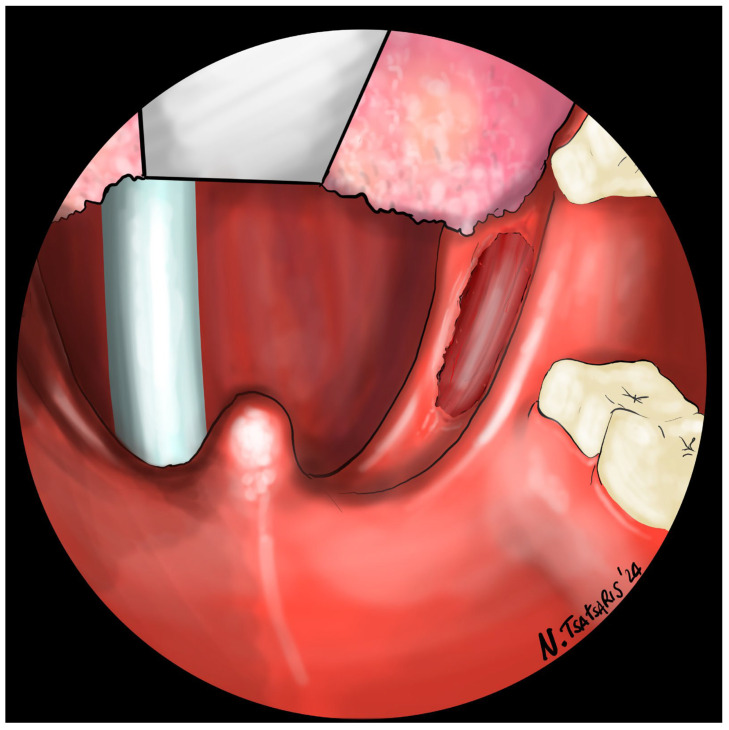
Schematic drawing of the field after tonsillectomy (right side).

The operation requires general anesthesia with nasotracheal intubation. If nasotracheal intubation is not possible, orotracheal intubation is also possible, but only if the base of the tongue is not to be operated on. Orotracheal intubation has no effect on the soft palate and the lateral oropharynx but makes it more difficult to treat the base of the tongue, which is partially covered by the tube. The patient is placed in the supine position, and a Boyle–Davis mouth gag is used to expose the oropharynx. Since exposure is one of the most important steps, I recommend taking time and trying different methods and different mouth gags if the surgeon is not satisfied with the exposure. At our facility, we use the Da Vinci XI robotic system (Intuitive^®^, Sunnyvale, CA, USA) with the rigid 0-degree endoscope. The first surgeon sits at the command station and controls the robotic arms, while the second surgeon sits at the patient’s head and helps clean the area, aspirate smoke, and assist with hemostasis. Typically, a Maryland dissector and a cautery spatula are used, but this depends on the surgeon’s preference and experience. The initial procedure involves bilateral tonsillectomy, if the tonsils are present, ensuring identification and protection of the palatopharyngeal muscles (PPM). For patients who have previously undergone tonsillectomy, a small bilateral excision of the mucosa covering the tonsillar region is performed to promote fibrous cicatrization of the muscles. The procedure is not described in detail, as it is the same as a standard tonsillectomy, except that it is performed with robotic arms by grasping and carefully dissecting the tonsils on both sides. The key point of the surgical procedure is to identify and carefully dissect the PPM in the middle of the tonsillar fossa (Figure 1, Figure 2, Figure 3, Figure 4 and Figure 5).

**Figure 6:**
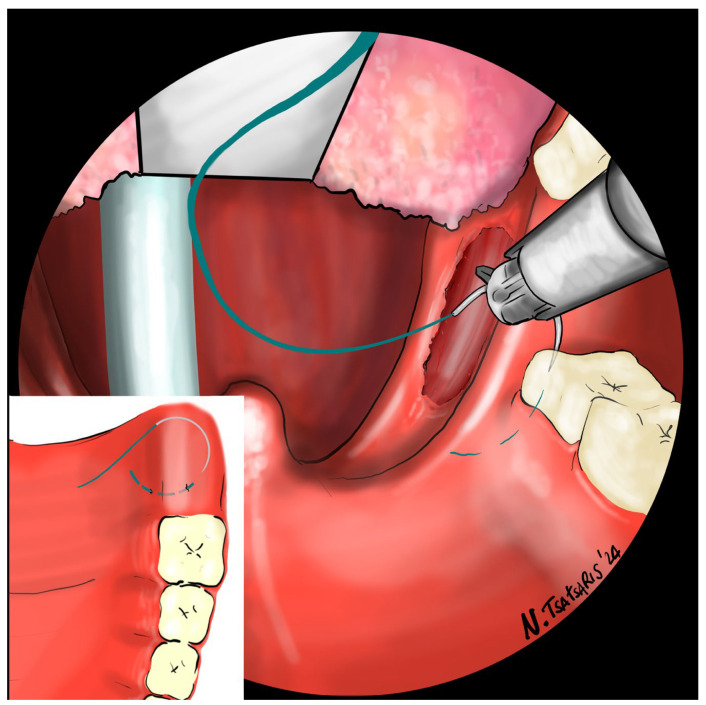
The needle is inserted with a self-locking barbed suture near the pterygomandibular fold, behind the last upper molar, including the periosteum of the maxillary tuberosity.

**Figure 7:**
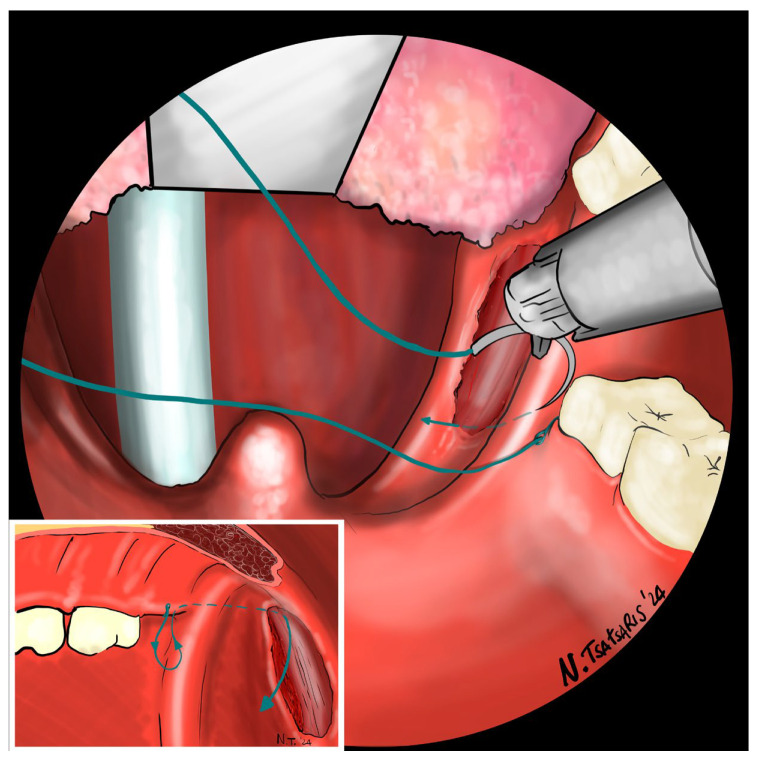
The needle is then inserted laterally into the PMF until it exits at the apex of the tonsillar fossa between the two tonsillar pillars.

**Figure 8:**
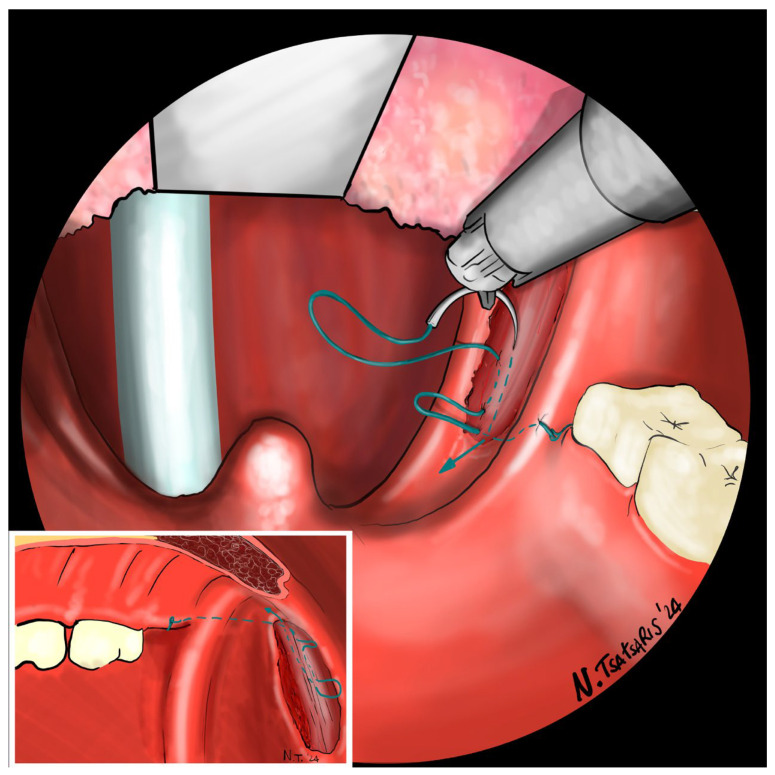
The needle is then driven through the PPM, sparing the mucosa of the posterior pillar. This step is repeated at least 3 times in different directions to elevate and lateralize the posterior pillar.

**Figure 9:**
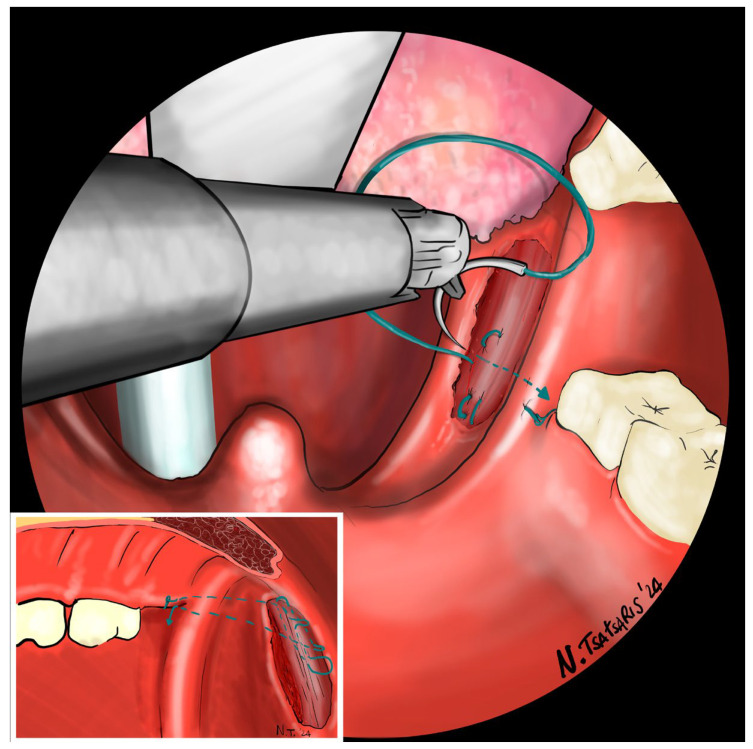
The needle is then re-inserted through the apex of the tonsillar fossa between the two pillars and guided laterally to the PMF.

**Figure 10:**
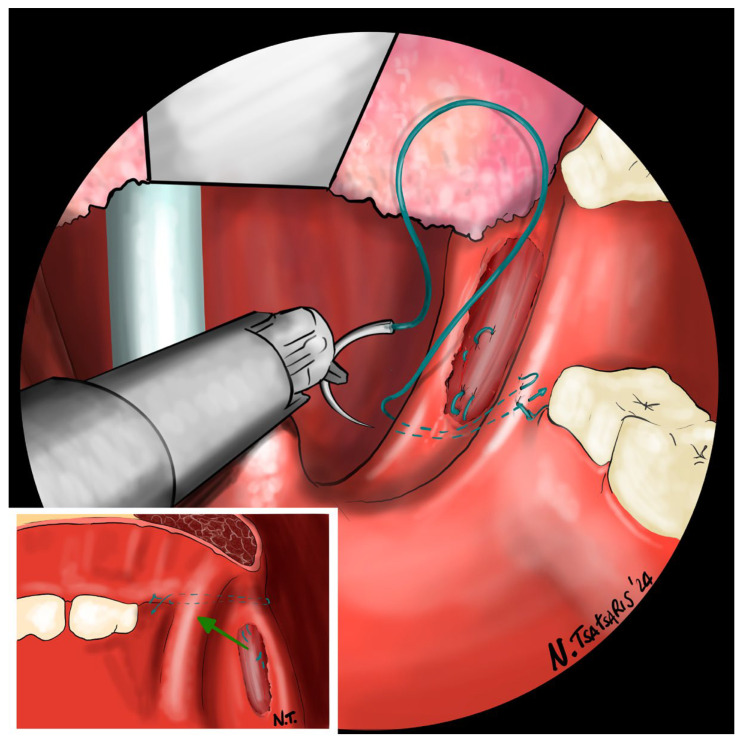
Finally, the suture encircles the posterior pillar, suspends it, and returns at a point medial to the PMF.

**Figure 11:**
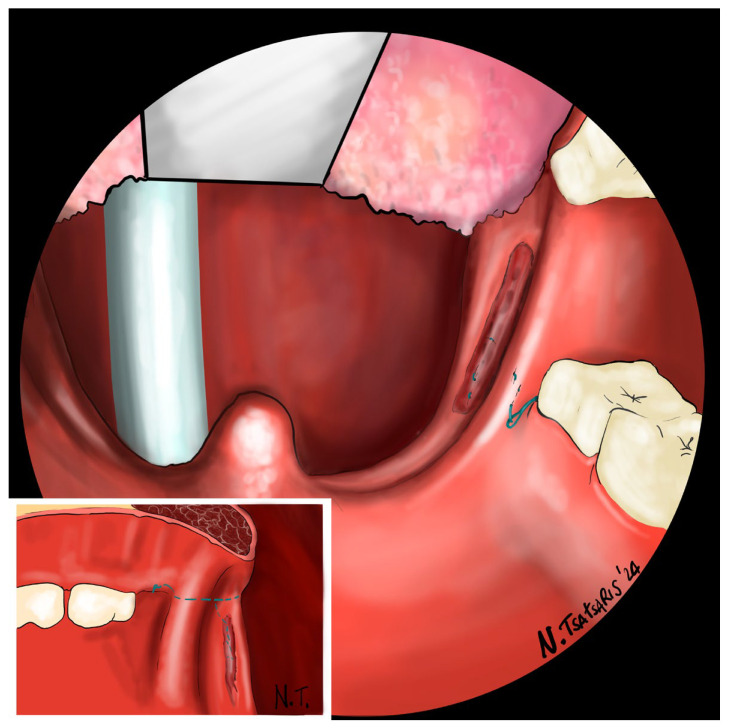
Proper tension of the suture achieves complete or partial closure of the tonsillar fossa and minimizes the risk of bleeding.

After the tonsillectomy is completed and hemostasis is finished, pharyngoplasty is performed. A robotic needle holder is now attached to the right robotic arm with a 2-0 self-locking barbed suture (V-Loc^TM^ 180, Covidien, Dublin, Ireland). The needle is inserted medial to the apex of the pterygomandibular fold (PMF), approximately behind the posterior upper molar, carefully including the periosteum of the maxillary tuberosity. A U-suture is created by hooking the end loop of the suture (Figure 6). Subsequently, the needle is laterally inserted into the PMF until it emerges at the apex of the tonsillar fossa between the two tonsillar pillars (Figure 7). The needle is then driven through the PPM, sparing the mucosa of the posterior pillar. This step is repeated at least three times in different directions to elevate and lateralize the posterior pillar (medial-to-lateral, lateral-to-medial, and again medial-to-lateral) (Figure 8). The needle is then re-inserted through the apex of the tonsillar fossa between the two pillars and guided laterally to the PMF (Figure 9). The flap can be pulled upwards and sideways, increasing the lateral diameter of the oropharynx and the stiffness of the lateral pharyngeal wall (LPW). The surgeon determines the appropriate tension by visually assessing the space gained, as the robotic approach does not allow for direct appreciation of tension in the wire. Finally, the suture encircles the posterior pillar, suspends it, and returns at a point medial to the palatopharyngeal muscle fold (PMF), as shown in Figure 10. Proper tension of the suture achieves complete or partial closure of the tonsillar fossa and minimizes the risk of bleeding (Figure 11). In the final step, the suture is closed with a horizontal U-shaped suture around the pterygoid hamulus. The barbed wire is pulled and cut close to the mucosa without the need for a knot. With sufficient tension on both sides, a three-dimensional widening of the velopharyngeal space is achieved. The operation takes approximately 1 h for both sides, including tonsillectomy.

Patients can start a soft oral diet on the first postoperative day and a normal diet after 2 weeks. They are instructed to rest until the tonsil regions are fully healed, as there is a risk of bleeding during the procedure, as with a normal tonsillectomy. Paracetamol is administered postoperatively to relieve the pain. Patients can return to a normal life after about 2 weeks.

## 3. Discussion

The described technique has been the standard of care since 2024 for selected patients diagnosed with OSA through clinical and instrumental assessment. Suitable candidates for this approach should meet criteria such as having been diagnosed with moderate to severe apnea-hypopnea index (AHI) via polysomnography (PSG). Additionally, they should not have macroglossia or restricted mouth opening, which can limit the maneuverability of robotic arms and the exposure of the treatment area. As this is a preliminary technical note, we explicitly focus on pharyngoplasty and do not consider combined procedures such as BOT resection.

Imaging studies have established the role of the lateral pharyngeal wall (LPW) in the pathogenesis of obstructive sleep apnea (OSA), with drug-induced sleep endoscopy (DISE) confirming that LPW narrowing is a significant independent risk factor for OSA [4,7,9]. Consequently, recent advancements in pharyngoplasty techniques have centered on widening and stabilizing the pharyngeal airspace by addressing LPW collapse rather than excising redundant pharyngeal soft tissue. Barbed sutures are designed to distribute tension uniformly along their entire length and generate dynamic vectors within the soft tissue without requiring knots or causing ischemic sequelae. Based on our observations, suture material exposure is unlikely. However, partial extrusion of the suture end due to tissue retraction may occur as a minor complication, which can be managed in outpatient settings.

Our technique is a variant of FEP, which was described in 2018 with the introduction of robot-assisted surgery and the non-resective strategy in PPM [9,10]. PPM plays a key role in the swallowing process. The wide distribution of its fibers suggests that it not only elevates the hyo-laryngeal complex but also functions as a nasopharyngeal sphincter in closing the pharyngeal isthmus. Therefore, the risk of dysphagia in older age would increase with the weakening of the PPM.

The main advantage of transoral robotic functional expansion pharyngoplasty is that it can be combined with other robotic procedures, for example, at the base of the tongue, as we know that OSA is rarely caused by an obstruction in a single level. At our institution, surgery for OSA usually targets more than one structure, and TORS is the preferred surgical technique for oropharyngeal surgery. Other advantages include the more precise and magnified 3D high-resolution view and finer movements that can help the surgeon to better preserve the posterior pillar during tonsillectomy. The use of robotic arms does not obstruct the view during the traction of the suture. This allows the surgeon to observe in real time the tension applied to achieve the desired expansion of the pharynx. Conversely, with the traditional technique, the surgeon’s hand may obscure the view, resulting in suture tension without a clear visual field and necessitating greater reliance on tactile memory. Since the procedure can be seen on the screen or even at the second surgical station, it can also be more helpful in a teaching facility, considering that the procedure can be followed by students, residents, or inexperienced doctors.

The main drawback is the lack of tactile feedback, which makes it difficult for the surgeon to understand how much tension to apply to the sutures. Iannella et al. demonstrated that muscle tissue handling and damage are crucial in pharyngoplasty [11]. Various techniques impact muscle tension and fiber tearing differently. Future robotic platforms could provide real-time data to surgeons about the tension forces applied during manipulation. Moreover, this is partially compensated for by the high resolution of the images, which provide visual feedback on how much tension the tissue is under and how dilated the oropharynx is. In this situation, barbed sutures can be helpful, as the suture itself does not need to be knotted. In our experience, this procedure does not take longer than the classic procedure.

Other disadvantages are the cost of robotic surgery and the still limited spread of robotic surgery, which may limit the spread of robotic procedures. These disadvantages will be partially overcome by the expected cost reduction in the near future and the continuous technological development of robotic devices. Preliminary results indicate that operative times and complication rates are comparable to Sorrenti’s technique.

The primary objective of this article is to introduce and detail the technique employed as the gold standard procedure at our facility, which involves robotic transoral surgery. This specific approach has not yet been documented in the literature and may represent a variation in the techniques outlined by Sorrenti et al. Ongoing studies are assessing its efficacy to determine the cost-effectiveness of this procedure in comparison to the standard techniques most commonly discussed in the literature.

## 4. Conclusions

Our technique is a variant of the previous techniques described in 2018. Robotic FEP may offer a viable option for surgically addressing the soft palate and lateral wall of the oropharynx in patients with OSA. This approach appears to be both easily learnable and reproducible. Further studies are needed to evaluate cost-effectiveness and long-term outcomes compared to classic techniques.

## Data Availability

All data used are available within this article.

## References

[1] Lv R., Liu X., Zhang Y., Dong N., Wang X., He Y., Yue H., Yin Q. (2023). Pathophysiological mechanisms and therapeutic approaches in obstructive sleep apnea syndrome. Signal Transduct. Target. Ther..

[2] Antonaglia C., Passuti G. (2022). Obstructive sleep apnea syndrome in non-obese patients. Sleep. Breath..

[3] Gulotta G., Iannella G., Vicini C., Polimeni A., Greco A., de Vincentiis M., Visconti I.C., Meccariello G., Cammaroto G., De Vito A. (2019). Risk Factors for Obstructive Sleep Apnea Syndrome in Children: State of the Art. Int. J. Environ. Res. Public. Health.

[4] Schwab R.J., Pack A., Gupta H.B., Metzger L.J., Oh E., Getsy J.E., Hoffman E.A., Gefter W.B. (1996). Upper airway and soft tissue structural changes induced by CPAP in normal subjects. Am. J. Respir. Crit. Care Med..

[5] Piccin O., Caccamo G., Pelligra I., Sorrenti G. (2022). Predictors of response to sleep apnea surgery addressing the lateral pharyngeal wall collapse. Am. J. Otolaryngol..

[6] AHan S., Cha H., Yang S.K., Kim S.Y., Han D.H., Kim D.Y., Rhee C.S., Kim H.J. (2023). Sleep parameter characteristics of patients with OSA who have retropalatal circumferential narrowing and the clinical significance of lateral pharyngeal wall collapse during sleep. Sleep. Breath..

[7] Cahali M.B. (2003). Lateral pharyngoplasty: A new treatment for obstructive sleep apnea hypopnea syndrome. Laryngoscope.

[8] Cammaroto G., Stringa L.M., Zhang H., Capaccio P., Galletti F., Galletti B., Meccariello G., Iannella G., Pelucchi S., Baghat A. (2020). Alternative Applications of Trans-Oral Robotic Surgery (TORS): A Systematic Review. J. Clin. Med..

[9] Sorrenti G., Piccin O. (2013). Functional expansion pharyngoplasty in the treatment of obstructive sleep apnea. Laryngoscope.

[10] Sorrenti G., Pelligra I., Albertini R., Caccamo G., Piccin O. (2018). Functional expansion pharyngoplasty: Technical update by unidirectional barbed sutures. Clin. Otolaryngol..

[11] Iannella G., Magliulo G., Di Luca M., De Vito A., Meccariello G., Cammaroto G., Pelucchi S., Bonsembiante A., Maniaci A., Vicini C. (2020). Lateral pharyngoplasty techniques for obstructive sleep apnea syndrome: A comparative experimental stress test of two different techniques. Eur. Arch. Otorhinolaryngol..

